# Mediterranean diet score linked to cognitive functioning in Czech women: a cross-sectional population-based study

**DOI:** 10.1007/s00394-025-03752-4

**Published:** 2025-07-12

**Authors:** Eliska Hrezova, Denes Stefler, Nadezda Capkova, Helena Vaclova, Martin Bobak, Hynek Pikhart

**Affiliations:** 1 RECETOX, Faculty of Science, Masaryk University, Kamenice 753/3, D29, 625 00 Brno, Czech Republic; 2 Department of Epidemiology & Public Health, Institute of Epidemiology and Health Care, University College London, London, WC1E 7HB England; 3 The National Institute of Public Health, Šrobárova 49/48, 100 00 Praha, Czech Republic; 4 Department of Public Health, Faculty of Medicine, Masaryk University, 625 00 Brno, Czech Republic

**Keywords:** Cognition, Cognitive decline, Mediterranean, Diet, Dietary habits, Aging

## Abstract

**Purpose:**

The evidence suggests that adherence to the Mediterranean diet (MD) may be beneficial in preventing cognitive decline. We aimed to explore this association in a Central European population.

**Methods:**

A total of 6,028 men and women from the Czech arm of the HAPIEE study were included in the analysis. Dietary data were collected using a food frequency questionnaire, and MD score (MDS) was calculated based on nine food groups. The MDS (range 0–17 points) was classified into three groups: low (0–7), medium (8–10), high (11–17). Cognitive function was measured using four tests assessing verbal memory and learning, verbal fluency, and attention, mental speed and concentration, and composite score, each of them converted to z-score. The associations between MDS and cognitive function were analyzed using multivariate linear regression in men and women.

**Results:**

There were no significant associations in men. By contrast, women with a dietary score of 8–10 points (B = 0.05, 95% CI: -0.002; 0.097), and those with a score of 11–17 points (B = 0.08, 95% CI: 0.016; 0.140) had a higher composite cognitive score than women in lowest adherence group. Regarding specific domains, women in the highest adherence group had significantly better immediate verbal memory (B = 0.12, 95% CI: 0.031; 0.205) and delayed recall (B = 0.12, 95% CI: 0.027; 0.212), respectively, than those in the lowest adherence group.

**Conclusion:**

Higher adherence to the MDS was associated with better cognitive functioning in verbal memory and composite cognitive score in Czech women. The results suggest that the Mediterranean diet may help to improve cognitive functioning.

**Supplementary Information:**

The online version contains supplementary material available at 10.1007/s00394-025-03752-4.

## Introduction

With the global population ageing, the prevalence of age-related diseases, particularly dementia and cognitive decline, is expected to rise significantly [1]. Dementia, characterized by a progressive decline in cognitive function, currently lacks effective treatment options [2]. Therefore, identifying disease-modifying risk factors to prevent cognitive decline has become a crucial area of research. Among these factors, nutrition, and specifically dietary patterns, has gained significant attention as a potential strategy to slow cognitive decline [3–5].

The Mediterranean diet (MD) is a dietary pattern with many associated health benefits [6]. MD emphasizes the consumption of whole grains, fruits, vegetables, legumes, nuts, and seeds. It includes moderate amounts of fish and minimal quantities of red and processed meats. Characteristic features of the MD include the use of olive oil as the primary cooking fat and the consumption of wine in small amounts with meals [7]. The MD ensures an optimal intake of essential nutrients that support brain health and help maintain the cognitive functions [8].

Historically, Mediterranean populations rank among the healthiest in the world, leading to the hypothesis that their traditional diet contributes to longevity [9]. Based on this, the MD has been widely studied in epidemiological research across different populations. Most prospective observational studies indicate that higher adherence to the MD is associated with slower declines in various cognitive domains and a reduced risk of dementia [10], in both Mediterranean [11, 12] and non-Mediterranean populations [13, 14]. However, findings from meta-analyses and randomized controlled trials (RCTs) are mixed. Some studies have shown beneficial effects of the MD on better cognitive outcomes [15–19], while others show no significant effects [20], especially in RCTs [21]. Despite this, large RCTs, such as those supplementing the MD with olive oil or nuts, report promising results [22, 23].

In this context, our study aims to explore the relationship between adherence to the Mediterranean diet and cognitive function among Czech older adults from the Health, Alcohol and Psychosocial Factors in Eastern Europe (HAPIEE) study.

## Methods

### Study design and sample

We used data from HAPIEE international prospective cohort study. Data collection for the Czech arm of the study began with the first wave between 2002 and 2005, during which a total of 8,856 individuals aged 45–69 years were enrolled from seven cities (Hradec Králové, Jihlava, Havířov, Karviná, Kroměříž, Liberec, and Ústí nad Labem). Participants underwent medical examination and completed a questionnaire survey [24]. The second wave took place between 2006 and 2008, with 5,210 individuals from the original sample participating. Informed consent was obtained from all participants, and the study was approved by the University College London Ethics Committee and The National Institute of Public Health in the Czech Republic.

### Dietary assessment

To assess dietary intake, we used semi-quantitative food frequency questionnaire (FFQ) with 136 food and drinks items [25]. Participants indicated how frequently consumed selected foods and drinks on nine-point frequency scale (from never or less than once a month to six or more times a day) over the past 3 months. Evaluation of the daily intake of different food groups was calculated using the EFSA food classification system [26], to calculate nutrient intake levels, the McCance and Widdowson Food Composition Database was used [27]. The used FFQ was validated on a randomly selected smaller sample of participants [28], estimating the correlation between consumption of fruit and vegetable and plasma biomarkers (vitamin C and beta carotene).

The calculation of Mediterranean diet score (MDS) in this study was described previously [29], and followed the method used by Sofi et al. [30]. Briefly, MDS consists of nine components with scoring system 0, 1 or 2 points for each component (Table 1). Olive oil usage had to be modified and was scored as 0 points for those reported usage of any other type of oil for cooking and 1 point for those who cook with olive oil. Finally, the MDS ranged from 0 to 17 points.

**Table 1:** Scoring criteria of the MDS components and the percentage of participants with maximum component scores

Components	Scoring criteria of MDS components			% of participants with maximum component scores	
	0 point	1 point	2 points	Men (*N* = 2,370)	Women (*N* = 3,297)
Vegetables (g/day)	< 100	100–250	> 250	22.8	36.9
Fruits and nuts (g/day)	< 150	150–300	> 300	38.6	60.4
Legumes (g/week)	< 70	70–140	> 140	20.3	19.4
Cereals (g/day)	< 130	130–195	> 195	65.4	58.1
Fish (g/week)	< 100	100–250	> 250	13.5	15.9
Meat and meat products (g/day)	> 120	80–120	< 80	18.2	31.7
Dairy products (g/day)	> 270	180–270	< 180	61.1	45.4
Alcohol (g/day)	> 24	< 12	12–24	9.4 (0.1)^a^	3.2 (0.1)^a^
Olive oil usage	Not used for cooking	Used for cooking	-	5.2 (4.7)^a^	5.5 (6.6)^a^

^a^% of missing data

### Cognitive outcomes

Cognitive function was assessed during first wave (2002–2005) on all non-working participants and on 20% random sub sample of working participants. During follow-up (2006–2008), cognitive function was examined in all participants by trained nurses using 4 neuropsychological tests. For the purpose of this cross-sectional analysis, only the first conducted examination was used for each participant, regardless of whether it was performed during the first or second wave (in order to exclude potential learning effect in repeated measurement). First, participants underwent a 10-word list recall task to evaluate verbal memory and learning. Immediate recall performance was measured by the total number of correctly recalled words across three 1-minute trials (scores ranging from 0 to 30). Delayed recall was evaluated after other cognitive tests were conducted (scores ranging from 0 to 10). Additionally, participants were asked to name as many animals as possible within 1 min to assessed verbal fluency. Finally, to assess attention, mental speed, and concentration, participants were instructed to identify and cross out 2 letters, P and W, within a random letter grid as quickly and accurately as possible within 1 min (scores ranging from 0 to 65).

### Covariates

Socio-demographic, lifestyle, and health-related variables were obtained from baseline questionnaires. Based on literature of hypothesized relationship, we included age, education, labour force participation (economic) and marital status, and material deprivation scale defined as the availability of enough money for food, clothing, and paying bills (each question with answers on Likert scale 0–4 with overall maximum deprivation score of 12). Additionally, physical activity, measured by the number of hours per week engaged in sports, games or hiking, smoking status, energy intake, and vitamin supplement intake were also considered as potential confounders. Baseline health status was measured by questions on previous heart attack/acute myocardial infarction, angina/ischemic heart disease, stroke, and cancer, and depressive symptoms were measured using the CES-D 20-item scale (Center for Epidemiologic Studies Depression Scale) [31].

### Analytical sample and multiple imputation of missing data

Only individuals with complete cognitive assessments, consisting of all four tests, were included in the analysis (*N* = 6,496). From these, individuals whose basal metabolic rate vs. reported energy intake ratio was in the top and bottom 1% of the distribution (*N* = 238) [32], and those who stated that the FFQ was not representative to their diet (*N* = 230) were excluded from the analysis. This resulted in dataset of 6,028 individuals. In wave 1, the sample consisted of 3,362 individuals (54% women), while in wave 2 there were 2,666 individuals (55% women). Within this analytical sample, missing covariates data (range 0.1-5.7%, Table 2), were imputed using the “mi imputed chained” command in STATA v 16.1. Age and sex were used as predictor variables. Presented results of regression analyses are pooled estimates of 50 imputed datasets.

### Statistical analysis

This study analyzed associations between MDS and cognitive outcomes. Single z scores (mean = 0, SD = 1) were computed for each cognitive tests to allow comparison between tests. As the main outcome of the analysis, a composite score of cognitive function was derived by taking the mean of the four z-scores. Adherence to MDS was classified as low (0–7 points), moderate (8–10 points) and high (11–17 points) adherence categories, following the approach used in previous study [29]. The association of MDS with cognitive function was modelled using multivariable adjusted linear regression with MDS treated as both categorical (with low MDS as reference category) and continuous variable. The results for the continuous models are presented as the change in cognitive score per one point increase in the MDS.

Firstly, we tested effect modification by adding interaction terms between MDS and sex, and education. Since there was a statistically significant interaction between MDS and sex (*p* < 0.001), the regression models were performed separately for males and females. There was no evidence for interaction with education (*p* = 0.576, *p* = 0.967 for males and females, respectively), therefore education was treated as a confounder. Adjustments were made for age in model 1; education (primary or less, vocational, secondary, university), economic status (working, not working), marital status (partnered, not partnered) and material deprivation scale were added in model 2. Finally, physical activity (no activity– 0 h per week, low– 1 to 2 h per week, mild– 3 to 5 h per week, and intensive activity– more than 6 h per week), energy intake (MJ/day), vitamin supplement intake (no intake, irregular– less than 3 times a week, regular intake– at least 3 times a week), smoking status (not current smoker, smoker), and presence of chronic diseases (yes, no) and CES-D were added to the model 3. To explore the robustness of our results and comparability between waves 1 and 2, we performed separate analyses for participants with cognitive data from wave 1 and those assessed for the first time in wave 2. To examine a possible effect of reverse causality, we conducted sensitivity analyses by excluding participants with history of stroke.

#### MDS-components and cognitive outcomes

Apart from the overall Mediterranean Diet Score, we also examined MDS components separately in order to assess the impact of individual dietary components; the results are shown as the change in cognition per one standard deviation increase in component score. To evaluate the relative importance of each MDS component, we calculated MDS repeatedly, each time excluding one specific component. The excluded component was then included as a covariate (as a continuous variable), while the MDS without that specific component as the main exposure.

## Results

### Sample characteristics

Characteristics of the study sample across MDS categories are detailed in Table 2. High adherence to the Mediterranean diet was found in 19% of men and 26% of women. Among men, those who were employed, more physically active, had a higher mean energy intake, were non-smokers and did not take vitamin supplements demonstrated higher adherence to the Mediterranean diet. In women, factors associated with high MDS included higher level of education, being unemployed but in a partnership, experiencing less material deprivation, higher levels of physical activity with higher energy intake, being non-smokers, not taking vitamin supplements, and reporting lower levels of depressive symptoms. Regarding MDS components, over 60% of men scored maximum points in cereals and dairy products categories, while the highest proportion of high scores in women were in the fruits and nuts category (Table 1). The component with the lowest proportion of high scores (except for alcohol and olive oil usage) was fish intake, both in men (13.5%) and women (15.9%).

**Table 2:** Characteristics of the study sample by MDS categories

		Men				Women			
		MDS categories							
		MDS low (*n* = 1,085)	MDS moderate (*n* = 1,125)	MDS high (*n* = 521)	*P* value (trend)^a^	MDS low (*n* = 887)	MDS moderate (*n* = 1546)	MDS high (*n* = 864)	*P* value (trend)^a^
Age, mean (SD), years		59.2 (7.1)	59.7 (6.9)	60.2 (6.9)	0.004	58.3 (7.1)	58.3 (7.0)	58.7 (6.8)	0.206
Education (%)	Primary or less	61 (5.6)	50 (4.4)	23 (4.4)	0.267	176 (19.8)	249 (16.1)	119 (13.8)	< 0.001
	Vocational	433 (39.9)	490 (43.6)	236 (45.3)		286 (32.2)	473 (30.6)	238 (27.5)	
	Secondary	377 (34.7)	366 (32.5)	160 (30.7)		343 (38.7)	661 (42.8)	397 (45.9)	
	University	208 (19.2)	214 (19.0)	96 (18.4)		80 (9.0)	158 (10.2)	108 (12.5)	
	N/A	6 (0.6)	5 (0.4)	6 (1.2)		2 (0.2)	5 (0.3)	2 (0.2)	
Economic status (%)	Not working	460 (42.4)	507 (45.1)	251 (48.2)	0.020	463 (52.2)	832 (53.8)	493 (57.1)	0.022
	Working	614 (56.6)	608 (54.0)	261 (50.1)		422 (47.6)	706 (45.7)	360 (41.7)	
	N/A	11 (0.1)	10 (0.9)	9 (1.7)		2 (0.2)	8 (0.5)	11 (1.3)	
Marital status (%)	Not partnered	172 (15.9)	160 (14.2)	66 (12.7)	0.082	306 (34.5)	489 (31.6)	247 (28.6)	0.008
	Partnered	909 (83.8)	964 (85.7)	452 (86.8)		579 (65.3)	1051 (68.0)	614 (71.1)	
	N/A	4 (0.4)	1 (0.1)	3 (0.6)		2 (0.2)	6 (0.4)	3 (0.3)	
Material deprivation, mean (SD)		1.4 (2.2)	1.4 (2.1)	1.2 (2.0)	0.271	1.9 (2.5)	1.7 (2.2)	1.6 (2.2)	0.002
	N/A	15	9	13		9	14	12	
Physical activity (%)	No	364 (33.5)	290 (25.8)	133 (36.4)	< 0.001	323 (36.4)	385 (24.9)	149 (17.2)	< 0.001
	Low	196 (18.1)	214 (19.0)	83 (15.9)		170 (19.2)	322 (20.8)	172 (19.9)	
	Mild	230 (21.2)	228 (20.3)	114 (21.9)		147 (16.6)	341 (22.1)	194 (22.5)	
	Intensive	265 (24.4)	374 (33.2)	181 (34.7)		212 (23.9)	453 (29.3)	318 (36.8)	
	N/A	30 (2.8)	19 (1.7)	10 (1.9)		35 (3.9)	45 (2.9)	31 (3.6)	
Energy, mean (SD), MJ		8.1 (3.6)	9.2 (4.4)	9.9 (5.4)	< 0.001	7.5 (3.8)	8.4 (7.9)	9.3 (8.4)	< 0.001
	N/A	-	-	4		-	-	3	
Smoking status (%)	Current smoker	344 (31.7)	280 (24.9)	105 (20.2)	< 0.001	213 (24.0)	332 (21.5)	153 (17.7)	0.001
	Non-smoker	731 (67.4)	836 (74.3)	409 (78.5)		667 (75.2)	1205 (77.9)	701 (81.1)	
	N/A	10 (0.9)	9 (0.8)	7 (1.3)		7 (0.8)	9 (0.6)	10 (1.2)	
CES-D, mean (SD)		8.5 (6.8)	8.8 (7.2)	8.6 (7.4)	0.555	11.8 (9.8)	11.1 (8.9)	10.2 (8.2)	< 0.001
	N/A	55	49	46		42	80	58	
Supplement intake (%)^b^	Yes, regularly	179 (16.5)	224 (19.9)	119 (22.8)	< 0.001	238 (26.8)	506 (32.7)	336 (38.9)	< 0.001
	Yes, irregularly	228 (21.0	296 (26.3)	112 (21.5)		238 (26.8)	482 (31.2)	267 (30.9)	
	No	674 (62.1)	604 (53.7)	284 (54.5)		404 (45.5)	553 (35.8)	254 (29.4)	
	N/A	4 (0.4)	1 (0.1)	6 (1.2)		7 (0.8)	5 (0.3)	7 (0.8)	
Chronic diseases (%)^c^	No	858 (79.1)	886 (78.8)	401 (77.0)	0.638	713 (80.4)	1237 (80.0)	681 (78.8)	0.764
	Yes	207 (19.1)	220 (19.6)	103 (19.8)		140 (15.8)	261 (16.9)	139 (16.1)	
	N/A	20 (1.8)	19 (1.7)	17 (3.3)		34 (3.8)	48 (3.1)	44 (5.1)	
Immediate recall, mean (SD)		21.9 (3.5)	21.9 (3.6)	21.5 (3.7)		22.9 (3.5)	23.4 (3.4)	23.5 (3.5)	
Delayed recall mean (SD)		7.3 (1.8)	7.2 (1.8)	7.1 (1.8)		7.7 (1.7)	7.9 (1.7)	8.0 (1.7)	
Verbal fluency, mean (SD)		23.7 (6.7)	23.9 (7.1)	23.3 (6.5)		23.1 (6.3)	23.9 (6.3)	24.0 (6.3)	
Letter cancelation, mean (SD)		17.4 (4.7)	17.3 (4.5)	16.6 (4.7)		18.3 (4.4)	18.6 (4.6)	18.5 (4.7)	

^a^p values were calculated by logistic or linear regression, covariates were used as outcome and MDS categories as continuously treated exposure variables

^b^Yes regularly (at least 3 times per week), Yes irregularly (less than 3 times per week)

^c^Yes (1 and more chronic diseases)

### Gender differences in the MDS-cognition relationship

Men in the highest adherence group to the MDS surprisingly showed inverse associations with the composite cognitive score, as well as in individual cognitive domains. Most of the associations were not significant, except for the letter cancellation test, where men in the high adherence group (B = -0.133, 95% CI: -0.242, -0.025, *p* = 0.016) had a significantly lower score compared to those in the lowest adherence group in the fully adjusted model (Table 3). MDS treated as a continuous variable showed not significant associations (Table 4).

**Table 3:** Association between MDS diet as a categorical exposure and cognitive outcomes

	Men (*n* = 2,731)						Women (*n* = 3,297)				
		MDS moderate		MDS high		P trend	MDS moderate		MDS high		P trend
		B	(95% CI)	B	(95% CI)		B	(95% CI)	B	(95% CI)	
Immediate recall	Model 1	0.020	(-0.056, 0.096)	-0.007	(-0.124, 0.086)	0.947	0.134	(0.073, 0.212)*	0.242	(0.147, 0.332)*	< 0.001
	Model 2	0.024	(-0.052, 0.099)	-0.020	(-0.125, 0.084)	0.928	0.094	(0.025, 0.163)*	0.146	(0.062, 0.231)*	< 0.001
	Model 3	0.020	(-0.056, 0.096)	-0.026	(-0.133, 0.080)	0.828	0.075	(0.005, 0.145)*	0.118	(0.031, 0.205)*	0.007
Delayed recall	Model 1	-0.026	(-0.107, 0.055)	0.000	(-0.113, 0.112)	0.820	0.089	(0.014, 0.164)*	0.200	(0.109, 0.292)*	< 0.001
	Model 2	-0.024	(-0.103, 0.055)	-0.011	(-0.121, 0.099)	0.707	0.057	(-0.016, 0.130)	0.133	(0.044, 0.223)*	0.004
	Model 3	-0.023	(-0.103, 0.057)	-0.005	(-0.117, 0.107)	0.790	0.046	(-0.028, 0.121)	0.120	(0.027, 0.212)*	0.013
Verbal fluency	Model 1	0.041	(-0.042, 0.124)	0.016	(-0.099, 0.132)	0.570	0.115	(0.040, 0.189)*	0.205	(0.114, 0.296)*	< 0.001
	Model 2	0.043	(-0.036, 0.123)	0.002	(-0.109, 0.113)	0.686	0.068	(-0.002, 0.137)	0.102	(0.017, 0.187)*	0.016
	Model 3	0.029	(-0.051, 0.110)	-0.020	(-0.133, 0.093)	0.968	0.054	(-0.016, 0.125)	0.076	(-0.011, 0.164)	0.078
Letter cancelation	Model 1	0.010	(-0.069, 0.089)	-0.125	(-0.235, -0.015)*	0.084	0.051	(-0.027, 0.128)	0.076	(-0.019, 0.170)	0.107
	Model 2	0.016	(-0.060, 0.093)	-0.130	(-0.236, -0.024)*	0.072	0.024	(-0.053, 0.101)	0.022	(-0.072, 0.115)	0.622
	Model 3	0.016	(-0.061, 0.093)	-0.133	(-0.242, -0.025)*	0.068	0.013	(-0.065, 0.091)	-0.001	(-0.098, 0.097)	0.985
Composite score	Model 1	0.011	(-0.047, 0.069)	-0.029	(-0.110, 0.052)	0.661	0.098	(0.045, 0.152)*	0.180	(0.116, 0.246)*	< 0.001
	Model 2	0.015	(-0.039, 0.069)	-0.040	(-0.115, 0.035)	0.513	0.061	(0.011, 0.110)*	0.101	(0.041, 0.161)*	0.001
	Model 3	0.011	(-0.044, 0.065)	-0.046	(-0.122, 0.030)	0.404	0.047	(-0.002, 0.097)	0.078	(0.016, 0.140)*	0.012

MDS low used as a reference category

*p value < 0.05

Model 1: adjusted for age

Model 2: adjusted for age, education, economic status, marital status and material deprivation scale

Model 3:adjusted for age, education, economic status, marital status, material deprivation scale, physical activity, energy intake, vitamin supplement intake, smoking status, presence of chronic diseases and CES-D

Women with moderate (B = 0.047, 95% CI: -0.002, 0.097, *p* = 0.063) or high (B = 0.078, 95% CI: 0.016, 0.140, *p* = 0.013), adherence to the Mediterranean diet had higher composite cognitive score than women in the lowest adherence group after full adjustment, with a significant linear trend observed across MDS categories (p trend = 0.012) (Table 3). Looking at specific domains, women in the highest adherence group had significantly better immediate verbal memory (B = 0.118, 95% CI: 0.031, 0.205, *p* = 0.035) and delayed recall (B = 0.120, 95% CI: 0.031, 0.205, *p* = 0.008, p trend = 0.013), respectively, than those in the lowest adherence group. Also, there was positive associations between verbal fluency domain and MDS categories in model 1 and model 2, however attenuated by other covariates in fully adjusted model 3 (MDS high: B = 0.076, 95% CI: -0.011, 0.164, *p* = 0.088). MDS treated as a continuous variable showed significant results (Table 4) only with domain of immediate verbal memory (B = 0.016, 95% CI: 0.001, 0.030, *p* = 0.032) and on the border of significance with overall cognitive function (B = 0.010 95% CI: 0.000, 0.020, *p* = 0.051). When we took a relative importance of all components of the MDS into account, excluding the “legumes” component strengthen the association between MDS and composite score of cognitive function from B = 0.010 (95% CI: 0.000, 0.020, *p* = 0.051) to B = 0.019 (95% CI: 0.007, 0.031, *p* = 0.002) in the fully adjusted model. The relative effect of the remaining components of the MDS was negligible.

**Table 4:** Association between MDS diet as a continuously treated exposure variable and cognitive function

	Men			Women		
	B*	(95% CI)	*P* value	B*	(95% CI)	*P* value
Immediate recall	0.001	(-0.015, 0.016)	0.939	0.016	(0.001, 0.030)	0.032
Delayed recall	-0.002	(-0.019, 0.014)	0.801	0.013	(-0.002, 0.028)	0.087
Verbal fluency	0.003	(-0.014, 0.020)	0.712	0.009	(-0.006, 0.023)	0.234
Letter cancelation	-0.014	(-0.030, 0.003)	0.099	0.003	(-0.013, 0.019)	0.730
Composite score	-0.003	(-0.014, 0.008)	0.606	0.010	(0.000, 0.020)	0.051

Adjusted for age, education, economic status, marital status, material deprivation scale, physical activity, energy intake, vitamin supplement intake, smoking status, presence of chronic diseases and CES-D

*Change in cognitive score per one point increase in the MDS

In the fully adjusted model 3 (Table S1), most associations between MDS components and composite cognitive function score were not significant in both men and women. A positive association was found with olive oil usage in women (B = 0.103, 95% CI 0.011, 0.194, *p* = 0.028) and cereal intake in men (B = 0.041, 95% CI 0.000, 0.081, *p* = 0.048). However, given the number of dietary components analyzed (*n* = 9), applying Bonferroni correction (adjusted significance threshold: *p* < 0.0056) resulted in none of these associations remaining statistically significant.

### Sensitivity analyses

We conducted separate analyses for participants with cognitive data from wave 1 and those assessed for the first time in wave 2 to account for potential bias related to the timing of cognitive testing. The results showed some variability across cognitive domains in women, with more consistent associations for immediate and delayed recall in women, and less consistent findings in domains such as verbal fluency and letter cancellation (Supplementary Tables S2). For the composite cognitive score, the direction of the association was similar in both waves, though the magnitude differed. An interaction test between wave and MDS for the composite score was non-significant in men (*p* = 0.475) and women (*p* = 0.504), indicating that timing of cognitive assessment did not materially modify the association. We therefore report pooled results as the primary analysis.

In sensitivity analyses excluding participants with a history of stroke, the associations between MDS and cognitive performance was slightly stronger compared to the primary analyses in women (Supplementary Table S3). This suggests that including individuals with a history of stroke may have attenuated the observed effects. There were no differences in men.

## Discussion

In this study, we investigated the association between diet and cognitive functioning using MDS and various cognitive domains. We found that higher adherence to the MDS was associated with better cognitive functioning in verbal memory, immediate and delayed, and composite cognitive score in Czech women. In contrast, we found not statistically significant associations in men, except for inverse associations within attention, mental speed, and concentration test. Overall, higher adherence to the MDS was lower in men than women.

The published literature addressing gender differences in the relationship between the MD and cognition is sparse and presents mixed findings. Some studies suggest that MD adherence is more strongly linked to cognitive benefits in women. For instance, higher MDS adherence has been associated with better cognitive status in women [33]. Similarly, cognitive status, including verbal memory, was positively associated with MD adherence, though no significant link was found with cognitive decline [34]. Small RCT found more pronounced cognitive benefits in women following an MD supplemented with coconut oil compared to men [35]. However, in women at higher risk of cognitive decline, adherence to the Mediterranean diet was not linked to cognitive changes over the following 5 and 9 years [36, 37]. In contrary to our results, significant associations between MD and increased score of animal fluency scores were found in men but not in women [38]. Another study indicated a beneficial effect of the MD on subjective cognitive function decline in men [39], while a Greek study reported a protective effect of MD on cognitive impairment in men, but not in women [40].

Several potential explanations for these gender differences are worth considering. Firstly, gender is a key social determinant of health that shapes health behaviors in complex ways. Women are generally more likely to select healthier food options [41], which may contribute to their higher adherence to the Mediterranean diet [42]. Conversely, men tend to prefer more foods with higher fat content or red meat [43, 44]. Men also faced difficulties in recalling what and how much they ate [45], leading to potential inaccuracies in FFQ completion. Completing an FFQ is a cognitively demanding task and considering that the average scores of all cognitive tests among men tended to decline across MDS adherence categories, it is possible that the absence of a relationship between MDS adherence and cognition in men could be influenced by a combination of these factors. In contrast, women performed better on tests of verbal memory. Females tend to have an advantage over males in verbal memory tests [46]. This advantage could partly explain the observed associations in women.

Additionally, the socio-economic context of the period when the data were collected is also crucial. The data reflect a time when the Czech Republic was undergoing significant socio-economic transformation from a command economy to a market-oriented economy [47]. Although the MD is not a typical dietary pattern for the Czech population, the opportunity to travel, exposure to different cultures and the introduction of new foods may have contributed to its increased inclusion in their diets, particularly in women.

Furthermore, a study participant´s age, ranging from 45 to 69 years in the first wave, represent a period where health concerns become more prominent. According to one Australian study, older males and females were more likely to have regular health check-ups. At the same time, men in the same age group were less interested in seeking information regarding illnesses such as obesity as women [48]. Thus, women might be more willing to adapt their lifestyle and dietary habits in response to health recommendations, potentially explaining their better adherence to the MD and the observed positive cognitive outcomes.

Overall, these findings suggest that socio-economic factors, gender-specific behaviours, and age-related health considerations all contribute to the observed gender differences in the relationship between Mediterranean diet adherence and cognitive function.

Beyond these differences, the MD´s neuroprotective effects are driven by its rich composition of bioactive nutrients, which influence multiple physiological pathways. Key mechanisms include reducing oxidative stress and inflammation through antioxidants (e.g., polyphenols, vitamins C and E), supporting vascular health, and modulating neuronal signalling [8]. Given the strong link between cerebrovascular health and cognitive function [49, 50], these mechanisms may partly explain our sensitivity analyses results. Specifically, excluding individuals with a history of stroke led to a slightly stronger associations between MDS and cognitive performance, suggesting that cerebrovascular conditions may attenuate the observed effects. This attenuation is likely due to both the direct impact of stroke on cognitive function and potential dietary changes following diagnosis. Since the MD is hypothesized to protect cognition primarily through vascular and inflammatory pathways, including individuals with a history of stroke in the main analysis remains justified. However, our sensitivity analysis further supports the robustness of our findings and highlights the role of vascular health in the diet-cognition relationship.

### Strengths and limitations

Our study involved a relatively young population in context of cognitive decline that may become more pronounced in later years. Although we tested differences between individuals younger and older than 60 years, the differences were negligible. Additionally, as mentioned earlier, the MD is not a typical dietary pattern for the Czech population. It is possible that another type of diet or scoring system might better explain the relationship between diet and cognition. We tested the Panagiotakos Mediterranean diet score [51], which incorporates additional components, such as potatoes, and is based on portion intake rather than grams. The correlation between the two scores was high (*r* = 0.52), and results were consistent with those from the Sofi index—no significant associations were observed in men, while in women, associations were slightly stronger but remained in the same direction. We also tested the Mediterranean-DASH Diet Intervention for Neurodegenerative Delay (MIND) diet, introduced by Morris et al. [52] but it showed no significant results in men, and the results in women were attenuated in fully adjusted models by other variables. In addition, we examined a traditional Eastern European diet pattern, reflecting rural dietary habits from the 1950–1960s [53]. Despite its historical relevance, this diet showed no significant associations with cognitive outcomes, either protective or harmful.

There are several weaknesses of this study. First, we relied on self-report of food consumption, particularly in relation to the amounts consumed. It is likely that this introduced misclassification of food intake data. As mentioned above, this misclassification may be more pronounced among men, which may partly explain the lack of association between MD and cognition in men. Further, FFQ was administered only once during the first wave of the study. This approach did not account for seasonal variations in food consumption and changes in the diet. Additionally, the food groups represented in the FFQ may not have accurately reflected a Mediterranean-type diet in populations outside this region.

Second, the cross-sectional design prevents establishing causality. While our findings suggest an association between adherence to the MD and cognitive function, we cannot determine the direction of this relationship. Reverse causality remains a possibility, particularly in individuals with pre-existing health conditions, who may have altered their diet post-diagnosis. However, for many adults, dietary habits tend to remain relatively stable over time [54].

Third, there was a time delay between dietary and cognitive assessments for a part of the study sample. To address this issue, we performed separate analyses for participants assessed in wave 1 and those assessed for the first time in wave 2. The results from both subgroups were generally consistent in terms of direction and magnitude, suggesting that the time lag between dietary and cognitive assessment did not materially affect the findings.

Finally, cognitive function is influenced by various factors, with diet being just one of them among a complex of healthy behaviours. Although we adjusted for a range of factors, we cannot exclude residual confounding, mainly due to other social and environmental determinants.

This is, to our knowledge, the first study from the Central European region to evaluate the relationship between the MD and cognition using a population-representative study. However, since the participants were from urban areas, the results may not be fully generalizable to the entire Czech population. The analysis was divided by gender, providing valuable insights given the distinctly different results between men and women. This highlights the importance of separate analyses to account for individual differences in adaptation to healthy behaviours, which are crucial in this age group.

## Conclusion

In summary, our study contributes valuable insights into the relationship between adherence to the Mediterranean diet and cognitive function among Czech older adults, particularly in the context of gender differences. While our findings indicate a positive association between higher MDS adherence and better cognitive performance in women, this relationship was not observed in men. Several factors may explain these results, including gender differences in dietary choices, reporting accuracy, and socio-cultural context. Our findings emphasize the complexity of dietary influences on cognitive health and the need for further longitudinal research to clarify these relationships, particularly in diverse populations.

## Electronic supplementary material

Below is the link to the electronic supplementary material.

Supplementary Material 1

## Data Availability

The datasets used and/or analyzed during the current study are available from the corresponding author on reasonable request.
