# Supplementary material for: Mediterranean diet score linked to cognitive functioning in Czech women: a cross-sectional population-based study
Source: Eur J Nutr. 2025 Jul 12;64(5):237. doi: 10.1007/s00394-025-03752-4 (PMC12255573; doi:10.1007/s00394-025-03752-4)
Supplement: Supplementary file 1 — Supplementary Material 1 [file 394_2025_3752_MOESM1_ESM.docx]

**Mediterranean diet score linked to cognitive functioning in Czech women: a cross-sectional population-based study**

European Journal of Nutrition

Eliska Hrezova, Denes Stefler, Nadezda Capkova, Helena Vaclova, Martin Bobak, Hynek Pikhart

Corresponding author

Eliska Hrezova

E-mail: [eliska.hrezova@recetox.muni.cz](mailto:eliska.hrezova@recetox.muni.cz)

RECETOX, Faculty of Science, Masaryk University, Brno 625 00, Czech Republic

**Table S1 Associations between MDS components and composite cognitive score**

|  | Men |  |  | Women |  |  |
| --- | --- | --- | --- | --- | --- | --- |
| Component | B* | (95% CI) | p value | B* | (95% CI) | p value |
| Vegetables | 0.012 | (-0.025, 0.048) | 0.527 | 0.025 | (-0.007, 0.056) | 0.127 |
| Fruits | -0.025 | (-0.057, 0.007) | 0.129 | 0.012 | (-0.019, 0.042) | 0.456 |
| Legumes | -0.028 | (-0.062, 0.005) | 0.099 | -0.021 | (-0.050, 0.007) | 0.143 |
| Cereals | 0.041 | (0.000, 0.081) | 0.048 | 0.016 | (-0.015, 0.047) | 0.313 |
| Fish | 0.001 | (-0.035, 0.038) | 0.938 | 0.023 | (-0.008, 0.053) | 0.142 |
| Meat and meat products | 0.002 | (-0.032, 0.035) | 0.921 | 0.019 | (-0.007, -0.046) | 0.155 |
| Milk and dairy | -0.012 | (-0.044, 0.020) | 0.471 | 0.005 | (-0.020, 0.031) | 0.679 |
| Alcohol | -0.013 | (-0.059, 0.032) | 0.560 | -0.012 | (-0.102, 0.077) | 0.786 |
| Olive oil usage | 0.090 | (-0.024, 0.203) | 0.121 | 0.103 | (0.011, 0.194) | 0.028 |

Adjusted for age, education, economic status, marital status, material deprivation scale, physical activity, energy intake, vitamin supplement intake, smoking status, presence of chronic diseases and CES-D .

* Change in cognitive score per one point increase in the MDS component

**Table S2 Association between MDS diet and cognitive outcomes, stratified by waves of cognitive assessment^a^ (fully adjusted model^b^)**

|  |  | Men (N w1=1,542; w2=1,189) | | | | | Women (N w1=1,820; w2=1,477) | | | | |
| --- | --- | --- | --- | --- | --- | --- | --- | --- | --- | --- | --- |
|  |  | MDS moderate | | MDS high | |  | MDS moderate | | MDS high | |  |
|  |  | B | (95% CI) | B | (95% CI) | p trend | B | (95% CI) | B | (95% CI) | p trend |
| Immediate recall | Wave 1 | 0.042 | (-0.061, 0.145) | 0.040 | (-0.105, 0.184) | 0.475 | 0.043 | (-0.052, 0.138) | 0.135 | (0.017, 0.253)* | 0.028 |
|  | Wave 2 | 0.003 | (-0.111, 0.116) | -0.077 | (-0.234, 0.080) | 0.440 | 0.122 | (0.019, 0.226)* | 0.109 | (-0.021, 0.239) | 0.072 |
|  | Pooled | 0.020 | (-0.056, 0.096) | -0.026 | (-0.133, 0.080) | 0.828 | 0.075 | (0.005, 0.145)* | 0.118 | (0.031, 0.205)* | 0.007 |
| Delayed recall | Wave 1 | 0.055 | (-0.051, 0.161) | 0.095 | (-0.055, 0.244) | 0.174 | 0.040 | (-0.059, 0.140) | 0.145 | (0.021, 0.270)* | 0.025 |
|  | Wave 2 | -0.100 | (-0.220, 0.020) | -0.082 | (-0.025, 0.084) | 0.179 | 0.059 | (-0.052, 0.170) | 0.096 | (-0.043, 0.235) | 0.167 |
|  | Pooled | -0.023 | (-0.103, 0.057) | -0.005 | (-0.117, 0.107) | 0.790 | 0.046 | (-0.028, 0.121) | 0.120 | (0.027, 0.212)* | 0.013 |
| Verbal fluency | Wave 1 | 0.017 | (-0.091, 0.126) | 0.044 | (-0.109, 0.198) | 0.571 | -0.007 | (-0.104, 0.089) | 0.040 | (-0.080, 0.160) | 0.555 |
|  | Wave 2 | 0.036 | (-0.088, 0.161) | -0.102 | (-0.273, 0.070) | 0.451 | 0.140 | (0.034, 0.244)* | 0.128 | (-0.003, 0.260) | 0.037 |
|  | Pooled | 0.029 | (-0.051, 0.110) | -0.020 | (-0.133, 0.093) | 0.968 | 0.054 | (-0.016, 0.125) | 0.076 | (-0.011, 0.164) | 0.078 |
| Letter cancelation | Wave 1 | -0.050 | (-0.156, 0.055) | -0.060 | (-0.156, 0.055) | 0.334 | 0.013 | (-0.090, 0.117) | 0.034 | (-0.096, 0.163) | 0.613 |
|  | Wave 2 | 0.105 | (-0.011, 0.220) | -0.210 | (-0.370, -0.050)* | 0.133 | 0.028 | (-0.091, 0.148) | -0.032 | (-0.181, 0.118) | 0.741 |
|  | Pooled | 0.016 | (-0.061, 0.093) | -0.133 | (-0.242, -0.025)* | 0.068 | 0.013 | (-0.065, 0.091) | -0.001 | (-0.098, 0.097) | 0.985 |
| Composite score | Wave 1 | 0.014 | (-0.060, 0.088) | 0.030 | (-0.039, 0.134) | 0.559 | 0.023 | (-0.044, 0.090) | 0.088 | (0.005, 0.171)* | 0.045 |
|  | Wave 2 | 0.008 | (-0.075, 0.091) | -0.133 | (-0.226, 0.003) | 0.133 | 0.102 | (0.027, 0.177)* | 0.078 | (-0.016, 0.172) | 0.067 |
|  | Pooled | 0.011 | (-0.044, 0.065) | -0.046 | (-0.122, 0.030) | 0.404 | 0.047 | (-0.002, 0.097) | 0.078 | (0.016, 0.140)* | 0.012 |

MDS low used as a reference category.

^a^ Results are presented separately for participants with cognitive data collected in wave 1 and for those with cognitive data collected in wave 2 (on average 3.6 years later)

^b^Adjusted for age, education, economic status, marital status, material deprivation scale, physical activity, energy intake, vitamin supplement intake, smoking status, presence of chronic diseases and CES-D

*p value <0.05

**Table S3 Sensitivity analyses of the association between MDS and cognitive outcomes excluding individuals with history of stroke at baseline**

|  | Men (N=2,539) | | | | | | Women (N=3,048) | | | | |
| --- | --- | --- | --- | --- | --- | --- | --- | --- | --- | --- | --- |
|  |  | MDS  moderate | | MDS high | |  | MDS  moderate | | MDS  high | |  |
|  |  | B | (95% CI) | B | 95% CI | P trend | B | (95% CI) | B | 95% CI | P trend |
| Immediate recall | Model 1 | 0.027 | (-0.054, 0.109) | -0.023 | (-0.135, 0.089) | 0.921 | 0.153 | (0.077, 0.229)* | 0.278 | (0.185, 0.371)* | <0.001 |
|  | Model 2 | 0.028 | (-0.049, 0.105) | -0.035 | (-0.141, 0.071) | 0.785 | 0.110 | (0.038, 0.181)* | 0.179 | (0.091, 0.266)* | <0.001 |
|  | Model 3 | 0.028 | (-0.050, 0.106) | -0.031 | (-0.140, 0.077) | 0.815 | 0.089 | (0.017, 0.161)* | 0.147 | (0.057, 0.237)* | 0.001 |
| Delayed recall | Model 1 | 0.007 | (-0.076, 0.090) | -0.009 | (-0.123, 0.105) | 0.944 | 0.106 | (0.028, 0.184)* | 0.227 | (0.132, 0.322)* | <0.001 |
|  | Model 2 | 0.006 | (-0.075, 0.087) | -0.020 | (-0.131, 0.092) | 0.817 | 0.075 | (-0.001, 0.151) | 0.156 | (0.063, 0.249)* | 0.001 |
|  | Model 3 | 0.008 | (-0.074, 0.091) | -0.010 | (-0.124, 0.105) | 0.944 | 0.064 | (-0.012, 0.141) | 0.142 | (0.046, 0.238)* | 0.004 |
| Verbal fluency | Model 1 | 0.058 | (-0.028, 0.144) | 0.024 | (-0.094, 0.142) | 0.434 | 0.116 | (0.038, 0.194)* | 0.203 | (0.109, 0.298)* | <0.001 |
|  | Model 2 | 0.057 | (-0.026, 0.140) | 0.012 | (-0.103, 0.126) | 0.528 | 0.070 | (-0.002, 0.143) | 0.096 | (0.008, 0.185)* | 0.027 |
|  | Model 3 | 0.040 | (-0.044, 0.125) | -0.013 | (-0.129, 0.104) | 0.891 | 0.059 | (-0.014, 0.132) | 0.074 | (-0.017, 0.166) | 0.095 |
| Letter cancelation | Model 1 | 0.035 | (-0.046, 0.117) | -0.116 | (-0.228, -0.003)* | 0.176 | 0.039 | (-0.041, 0.120) | 0.063 | (-0.036, 0.161) | 0.200 |
|  | Model 2 | 0.041 | (-0.038, 0.120) | -0.119 | (-0.228, -0.010)* | 0.163 | 0.013 | (-0.067, 0.093) | 0.005 | (-0.093, 0.103) | 0.899 |
|  | Model 3 | 0.040 | (-0.040, 0.121) | -0.119 | (-0.230, -0.008)* | 0.164 | 0.004 | (-0.077, 0.085) | -0.014 | (-0.115, 0.088) | 0.812 |
| Composite score | Model 1 | 0.032 | (-0.028, 0.092) | -0.031 | (-0.113, 0.051) | 0.810 | 0.103 | (0.048, 0.159)* | 0.193 | (0.125, 0.260)* | <0.001 |
|  | Model 2 | 0.033 | (-0.022, 0.089) | -0.040 | (-0.117, 0.036) | 0.658 | 0.067 | (0.016, 0.118)* | 0.109 | (0.047, 0.171)* | <0.001 |
|  | Model 3 | 0.029 | (-0.027, 0.085) | -0.043 | (-0.121, 0.034) | 0.579 | 0.054 | (0.003, 0.105)* | 0.087 | (0.023, 0.151)* | 0.006 |

MDS low used as a reference category.

* p value <0.05

Model 1: adjusted for age

Model 2: adjusted for age, education, economic status, marital status and material deprivation scale

Model 3: adjusted for age, education, economic status, marital status, material deprivation scale, physical activity, energy intake, vitamin supplement intake, smoking status, presence of chronic diseases and CES-D
